# Erratum to: Head/skull injury potential of empty 0.5-l beer glass bottles vs. 0.33-l Coke bottles

**DOI:** 10.1007/s00414-023-02962-x

**Published:** 2023-02-16

**Authors:** C Nentwig, S Steinhoff, J Adamec, SN Kunz

**Affiliations:** 1 Universität Ulm, Ulm, Germany; 2 Abteilung Für Radiologie, Bundeswehrkrankenhaus Ulm, Ulm, Germany; 3 Institut Für Rechtsmedizin, Ludwig-Maximilians-Universität München, Munich, Germany; 4 Institut Für Rechtsmedizin des Universitätsklinikums Ulm, Ulm, Germany

**Keywords:** Assault, Blast with an object, Forensic medicine, Glass bottles, Head injury

## Abstract

In the context of further impact tests with various striking weapons against the skull, it turned out that the manufacturer had incorrectly calibrated the force measuring plate, which was used in our earlier experiments. When the tests were carried out again under the same conditions, the measurement results were significantly higher.

In the context of further impact tests with various striking weapons against the skull, it turned out that the manufacturer had incorrectly calibrated the force measuring plate, which was used in our earlier experiments.

When the tests were carried out again under the same conditions, the measurement results were significantly higher (Tables 1 and 2).

**Table 1:** Measurement results, strikes with the side of 0.5-l beer bottles on pork rind—aluminium

Nr	Fracture (y/n)	Force (*N*)
1.1	n	5.979
1.2	n	6.521
1.3	n	3.388
2.1	n	6.145
2.2	y	7.730
3.1	y	4.099
4.1	n	5.658
4.2	y	3.054
5.1	n	4.481
5.2	n	5.214
5.3	y	2.078
6.1	n	3.833
6.2	n	4.726
6.3	y	5.688
7.1	y	2.927
8.1	n	5.718
8.2	y	3.353
9.1	y	3.383
10.1	n	5.094
10.2	n	5.630
10.3	y	5.911
11.1	n	8.382
11.2	y	6.479
12.1	n	8.265
12.2	n	5.121
12.3	y	3.787
13.1	n	6.110
13.2	y	5.718
14.1	n	6.631
14.2	y	2.270
15.1	n	7.059
15.2	y	4.600
16.1	n	5.323
16.2	y	2.975
17.1	n	4.522
17.2	n	6.644
17.3	y	3.961
18.1	n	3.996
18.2	y	3.415
19.1	n	4.492
19.2	y	2.327
20.1	n	3.017
20.2	y	1.785

**Table 2:** Measurement results, strikes with the side of 0.33-l Coke bottles on pork rind—aluminium

Nr	Fracture (y/n)	Force (*N*)
1.1	n	4.585
1.2	y	4.986
2.1	n	5.256
2.2	y	6.708
3.1	y	4.454
4.1	n	4.915
4.2	n	5.918
4.3	y	5.939
5.1	n	6.948
5.2	n	7.826
5.3	n	7.816
5.4	y	5.617
6.1	y	5.988
7.1	n	5.319
7.2	n	5.793
7.3	n	6.589
7.4	n	7.192
7.5	n	10.30
7.6	y	8.650
8.1	n	8.798
8.2	y	6.570
9.1	n	5.836
9.2	n	6.025
9.3	y	7.098
10.1	y	6.403
11.1	n	5.778
11.2	n	7.256
11.3	y	5.459
12.1	y	4.652
13.1	n	7.415
13.2	n	8.642
13.3	y	6.498
14.1	n	7.514
14.2	y	5.180
15.1	n	6.242
15.2	y	6.151
16.1	y	4.032
17.1	n	7.001
17.2	n	7.916
17.3	y	6.299
18.1	n	6.069
18.2	y	5.495
19.1	y	4.955
20.1	y	5.875

While the forensic-biomechanical basic conditions of hits with a glass bottle against the human skull remained the same, higher maximum values of the force transmitted were found. With a maximum force transfer of more than 8 kN for both blasts with a 0.5-l beer bottle as well as with a 0.33-l Coke bottle, fractures of the cranium are possible.

Our conclusions therefore need to be adjusted based on the likelihood of potential life-threatening injuries.

## Conclusion

The possible maximum impact force which can be transferred onto a human skull by blows made with an empty 0.5-l glass beer bottle is slightly lower (8.3 kN), but overall comparable with an empty 0.33-l Coke bottle (8.7 kN).

If the glass bottle comes into contact with the face (particularly the nasal bone, cheekbones), bone injuries are likely, and if the skull is hit, fractures are to be classified as possible. Thus, life-threatening blunt trauma injuries are possible in a healthy adult.

